# Juvenile dermatomyositis: clinical features, laboratory findings, treatment modalities and disease course (a single center experience)

**DOI:** 10.1186/1546-0096-12-S1-P276

**Published:** 2014-09-17

**Authors:** Ozgur Kasapcopur, Kenan Barut, Pinar Ozge Avar, Salim Caliskan, Lale Sever, Nil Arisoy

**Affiliations:** 1 Pediatric Rheumatology, Istanbul University, Cerrahpasa Medical Faculty, Istanbul, Turkey; 2 Pediatric Nephrology, Istanbul University, Cerrahpasa Medical Faculty, Istanbul, Turkey

## Introduction

Juvenile dermatomyositis (JDM) is an uncommon vasculitis in childhood. JDM is the most prevalent idiopathic inflammatory myopathies of childhood. JDM is characterized by proximal muscle weakness and typical skin involvement.

## Objectives

To describe demographics, clinical features, laboratory findings and treatment modalities of patients with juvenile dermatomyositis (JDM) at a referral pediatric rheumatology center in Turkey.

## Methods

Retrospective review of forty three patients meeting Bohan and Peter criteria diagnosed as JDM at the Pediatric Rheumatology Department of Istanbul University Cerrahpasa Medical Faculty between the years 2003-2013.

## Results

Forty three patients were identified; thirty of them (69.3%) were female, thirteen of them (%30.2) male. Mean follow-up period was 48 months (3-168). Mean age for the beginning of the disease was 6.3+/-4.4, mean age for the diagnosis was 6.9+/-4.4. Forty two patients (97.7%) had heliotropic rash and Gottron papules, 39 patients (90.7%) had muscle weakness, 36 patients (87.3%) had erythroderma and 16 patients (37.2%) had calcinosis as the most common clinical features of the disease. Forty one of the patients (95%) had elevated acute phase responses at presentation. Twenty seven of the patients (62.8%) had anti-nuclear antibody (ANA) positivity. Anti-jo 1 and antibodies to extractable nuclear antigen positivity were not found at all. Mean creatine kinase levels at presentation were 2245+/-3404 IU/L, median level was 564 IU/L. Mean time returning to normal levels was 6.6+/-17.8 months, median time was 3 months. Muscle biopsy was performed on 14 patients (32.6%) and all of them showed findings of inflammatory myopathy. Electromyography (EMG) was performed on 25 patients (58.1%) and all of them suggested inflammatory myopathy. All patients had been treated with corticosteroids at different dosages. Methotrexate was used in 41 patients (95.3%), at the mean dosage of 14.5+/-7.1 (7.5-40) miligrams per week and for the mean duration of 45+/-36 months. Cyclosporine was used in 16 patients (37.2%). In 13 of 16 patients with calcinosis (81.3%), alendronate was used. Muscle weakness was present in 39 patients (90.7%) in the beginning, mean duration for the recovery of muscle weakness was 7.9+/-10 months, median duration was 4 months. Only two patients on follow-up period could not achieve self-movement capability. Both were referred to our clinic after a long duration of the disease and had widespread calcinosis. There were no significant relationship between mean duration of the muscle enzymes to return normal values and mean duration of the disappearance of the muscle weakness (p=0.7).

## Conclusion

Juvenile dermatomyositis is a rare systemic vasculopathy; however, it is an important disease because it may cause severe muscle weakness and paralysis. JDM should be keep in mind by its presentation as muscle weakness and different skin rashes in early childhood. It should be differed from some kind of genetic muscle diseases. Methotrexate and prednisolone should be first choices for the treatment of JDM.

## Disclosure of interest

None declared.

